# A Novel Method for Online Extraction of Small-Angle Scattering Pulse Signals from Particles Based on Variable Forgetting Factor RLS Algorithm

**DOI:** 10.3390/s21175759

**Published:** 2021-08-26

**Authors:** Rongrui Zhang, Heng Zhao

**Affiliations:** Centre for Lidar Remote Sensing Research, School of Mechanical and Precision Instrument Engineering, Xi’an University of Technology, Xi’an 710048, China; jerry_zhang2020@foxmail.com

**Keywords:** optical particle counter, small angle, light scattering, particulate matter, adaptive filtering, recursive least squares

## Abstract

The small-angle optical particle counter (OPC) can detect particles with strong light absorption. At the same time, it can ignore the properties of the detected particles and detect the particle size singly and more accurately. Reasonably improving the resolution of the low pulse signal of fine particles is key to improving the detection accuracy of the small-angle OPC. In this paper, a new adaptive filtering method for the small-angle scattering signals of particles is proposed based on the recursive least squares (RLS) algorithm. By analyzing the characteristics of the small-angle scattering signals, a variable forgetting factor (VFF) strategy is introduced to optimize the forgetting factor in the traditional RLS algorithm. It can distinguish the scattering signal from the stray light signal and dynamically adapt to the change in pulse amplitude according to different light absorptions and different particle sizes. To verify the filtering effect, small-angle scattering pulse extraction experiments were carried out in a simulated smoke box with different particle properties. The experiments show that the proposed VFF-RLS algorithm can effectively suppress system stray light and background noise. When the particle detection signal appears, the algorithm has fast convergence and tracking speed and highlights the particle pulse signal well. Compared with that of the traditional scattering pulse extraction method, the resolution of the processed scattering pulse signal of particles is greatly improved, and the extraction of weak particle scattering pulses at a small angle has a greater advantage. Finally, the effect of filter order in the algorithm on the results of extracting scattering pulses is discussed.

## 1. Introduction

The mass concentration of particulate matter is often used as an important numerical index to evaluate regional air quality. Some studies have found that the number concentration of particles in some environments is positively correlated with the concentration of bioaerosol [1]. Fine particles can provide carriers for bioaerosols, and some particles smaller than 5 μm can be directly inhaled by the human body and cause damage to the respiratory system [2]. Some scholars have pointed out that areas with severe basic pollution have more deaths from COVID-19 [3,4]. Xie et al. showed that air pollution, such as PM2.5 and PM10, is positively correlated with the mortality of individuals infected with COVID-19 [5]. According to the 2020 EPA certification list published by the American Environmental Monitoring Technical Information Center (AMTIC) [6], the main methods for detecting the mass concentration of particles are the manual reference method, the manual equivalence method, and the automatic equivalence method. The most widely used is the automatic equivalence method, including the tapered element oscillating microbalance (TEOM) method [7], the β-ray method [8], and the light scattering method [9,10]. Compared with other measurement methods, the light scattering method has many advantages, including fast measurement speed, high precision, good repeatability, online and real-time non-contact measurement [11].

The optical particle counter (OPC) is a typical application based on the light scattering method to measure the mass concentration of suspended particles in the air. When a single particle passes through the photosensitive region, the scattering light signal is generated by the electromagnetic wave action of the particle [12]. Due to the different sizes and properties of the particles passing through, the output electrical signal is a series of pulse signals. The level of pulse signals can invert particle size information, and the number of pulses corresponds to the number of particles [13]. The detection angle of the traditional OPC is mostly 90°. In fact, at this daylighting angle, some darker aerosols with strong light absorption (e.g., soot, black carbon, dust) will scatter a tiny amount of light, about ten times less than some highly transparent particles (e.g., glass). In the measurement, some relatively dark particles will be ignored, and only brighter particles will be considered, which will cause measurement deviation. The traditional OPC is limited by the properties of the particles (complex refractive index and density). Before measurement, very complex calibration means are required to determine the parameters of the instrument [14]. At the same time, when the measurement environment is quite different from the calibration environment, the measurement results often lose the reference value. A small-angle OPC can detect particles with strong light absorption. In addition, measuring at a small angle can ignore the influence of particle properties and singly and more accurately determine the particle size. Compared with the traditional 90° receiving angle, this technique of forward small-angle detection will be more severely affected by stray light and background noise. For such a forward scattering angle, stray light may occupy about 80% of the entire signal. This seriously interferes with extracting the scattering pulse signal of the particles and obtaining the pulse height and pulse number. The traditional forward small-angle OPC usually evaluates the background noise level before the pulse signal appears in order to obtain the particle pulse signal [15]. It can also use the high-efficiency particulate air (HEPA) filter to obtain the zero-drift of the photodetector before each measurement and subtract the zero-drift value from each subsequent measurement result to obtain the scattering pulse signal of particles [16]. When detecting larger particle sizes (>1 μm), these traditional methods for extracting particle pulse signals have a good signal-to-noise ratio. However, when detecting particles below 1 μm, the scattering pulse signal of particles will be submerged in the background noise caused by the scattering light of air molecules, stray light from the light source, and electrical noise. Therefore, the effective extraction of particle scattering pulse signals of 1 μm size and below becomes key to improving the accuracy of the small-angle OPC detection.

In this paper, we propose a novel method for online extraction of small-angle scattering pulse signals from particles based on the variable forgetting factor (VFF) recursive least squares (RLS) algorithm. A VFF strategy is introduced to solve the problem of small-angle scattering pulse signals with varying particle sizes and light absorptions. By designing the forgetting factor of the RLS algorithm, the proposed method can adapt to the requirements of detecting different particle sizes and different light absorption particles and effectively suppress the forward scattering interference signal. Experimental results show that the proposed method can effectively extract the scattering pulse signals of particles smaller than 1 μm and can self-adapt to the dynamic changes of the scattering pulse under different particle sizes, which greatly improves the resolution of the pulse signal.

## 2. Principle of Adaptive Filtering Forward Scattering Cancellation

To remove the interference of the forward scattering of air molecules, direct radiation of the laser light source, and other system noise, the traditional method of forward scattering cancellation is used to calculate the level of the detection signal without particles and then subtract the detection signal with particles to obtain the pulse signal of particles. Theoretically, the background noise obtained with the same detection system in the same environment should be basically unchanged, and the influence of air molecular scattering can often be ignored. In practical applications, however, the direct radiation signal of the light source, the scattering signal of the air molecules, and the stray light noise obtained by the photodetector will be very different, because of subtle changes in the detection environment, the stability of the laser power, and the jitter of the signal processing time. When the particle detection signal is very weak, the forward scattering of the air molecules can no longer be ignored. These differences will seriously interfere with the extraction of the particle pulse signals, and traditional methods tend to submerge the pulse signals in noise. The principle of adaptive filtering to cancel forward scattering uses a reference signal without particle scattering pulses and a detection signal with particle scattering pulses as the two inputs to the filter. By adjusting the filter parameters, the forward scattering noise of the two parts can be adjusted adaptively and they will offset each other. The characteristics of the pulse signal can be highlighted in the actual detection to eliminate the forward scattering noise and extract the particle pulse signal.

The adaptive filter can be used as the model and inverse model of system signal transmission, so it can be used for the adaptive identification, the adaptive prediction, and the adaptive equalization. The identification algorithms as the core of adaptive filter mainly includes the stochastic gradient method, coupled identification method, auxiliary model method, Newton iterative method, multi-innovation method, least mean square method, and recursive least squares method [17,18,19,20]. For large-scale strong nonlinear systems with complex structure, the filtering technology combined with multi-innovation identification theory can significantly improve the filtering and identification accuracy [20]. The reconstruction of lost data is the basis of the signal modeling, the signal filtering, and the signal estimation [21,22]. The gradient-based parameter estimation algorithm uses the martingale convergence theorem and Kronecker lemma to reconstruct the lost data with a dynamic model for the irregular and sparse system [23]. Gopaluni studied the identification of missing observation data in the nonlinear process based on the maximum expectation algorithm and the particle filter method [24]. Cao models multi-sensor input data and uses fuzzy theory to process dynamic data to realize the identification and extraction of train control data features, which improves the system efficiency and robustness [25]. Ding proposed an iterative estimation algorithm based on gradient and least-squares to estimate the parameters of a multi-input multi-output system with colored auto-regressive moving average noise from the data [26]. In addition, Zhang used the Long and Short-Term Memory (LSTM) network model to make effective filtering and adaptive identification of the vibration signals of the fuselage of the quadcopter, so as to realize effective detection and analysis of blade faults [27]. The Least Mean Square method parameter estimation algorithm mainly include the stochastic gradient algorithm and the projection algorithm. The former cannot track time-varying functions, while the latter is sensitive to noise. Compared with the forgetting factor recursive least squares (RLS) algorithm, the projection algorithm can provide accurate parameter estimation for time-invariant deterministic systems, and requires less calculation [28]. The LMS algorithm is suitable for filtering and eliminating stationary random noise signals. In nonstationary environments, the tracking ability and convergence speed of the LMS algorithm for changing noise is not as good as the RLS algorithm. The least squares algorithm defines its criterion function to take the sum of the squares of error between the model output and the system output, and minimizes this criterion function to obtain the estimation of the unknown quantity of the system [29,30]. When the identified system is running, ach time new observation data is obtained, it will be corrected based on the previous estimation results [30]. Thus, new parameter estimates are obtained and the estimation error is reduced. In this way, with the successive introduction of new observation data, he parameter estimation is carried out one after another until the parameter estimation reaches a satisfactory degree of accuracy [29]. The principle of the least squares method is relatively simple and has good statistical characteristics under certain conditions, which can realize better identification of system characteristics [31]. The LMS algorithm is suitable for filtering and eliminating stationary random noise signals. In nonstationary environments, the tracking ability and convergence speed of the LMS algorithm for changing noise is not as good as the RLS algorithm [32,33]. Therefore, we used the RLS algorithm for adaptive filtering of the detection signal and improved the RLS algorithm according to the characteristics of the small-angle particle scattering signal.

**Figure 1:**
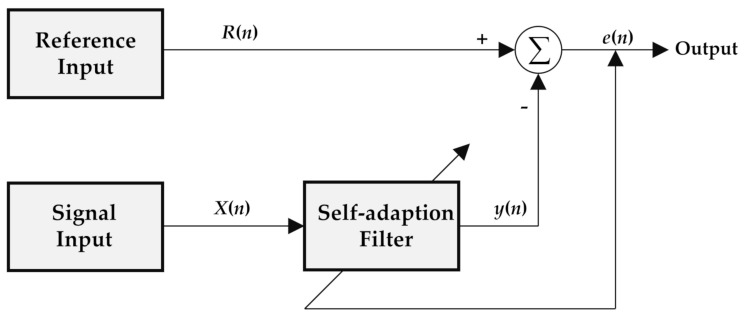
Block diagram of the adaptive filtering principle.

The signal *S* received by the small-angle OPC photodetector can be expressed by the linear superposition of several parts, as shown in Equation (1):

(1) $$S=S_{P}+S_{L}+S_{A}+S_{B}$$

where *S_P_* is the scattering signal of particles, *S_L_* is the diffraction signal of light source, *S_A_* is the scattering signal of air molecules, and *S_B_* is the system background noise. In the RLS algorithm proposed in this paper, *x*(*n*) is the detection signal with particle scattering pulse (*S_P_* + *S_L_* + *S_A_* + *S_B_*), and *r*(*n*) is the reference signal without particle scattering pulse (*S_L_* + *S_A_* + *S_B_*), and *y*(*n*) is the estimated signal of *x*(*n*) after adaptive filtering. The output error signal *e*(*n*) is the best estimate of the particle scattering pulse signal. As opposed to the LMS algorithm, the RLS algorithm uses the sum of square errors of the exponentially weighted factors as the cost function, and its expression is:

(2) $$ℑ(n)=\sum_{i=0}^{n}\lambda^{n-i}{\left[r(i)-\omega^{T}(n)x(i)\right]}^{2}$$

where *λ* is the weighting factor called the forgetting factor (0 < *λ* ≤ 1). As the number of iterations increases, the previous data will be gradually forgotten. *x*(*n*) = [*x*(*n*) + *x*(*n* − 1) + *x*(*n* − 2) +, …, *x*(*n* − *L* + 1)] is the input vector of the detection signal with particle scattering pulses, *L* is the filtering order, *r*(*i*) is the input vector of the reference signal without particle scattering pulses, *ω*(*n*) is the vector of iterative weight coefficients, and *ω*^T^(*n*) represents the transpose of *ω*(*n*). A set of iterative formulas for RLS can be derived as follows [34]:

(3) $$\begin{array}{l} \omega (n)=\omega (n-1)+k(n)e(n)\\ k(n)=\frac{P(n-1)x(n)}{\lambda +x^{T}(n)P(n-1)x(n)}\\ P(n)=\frac{1}{\lambda }[P(n-1)-k(n)x^{T}(n)P(n-1)]\\ e(n)=r(n)-y(n)=r(n)-\omega^{T}(n-1)x(n) \end{array}$$

where *k*(*n*) is the vector of Kalman gain and *P*(*n*) is the inverse matrix of the autocorrelation matrix. The initialization values of the algorithm are *ω*(0) = 0, *P*(0) = *δ*^−1^*I*, where *I* is the identity matrix and *δ* is the regularization parameter. In the iterative calculation process, the weight vector and gain vector are constantly updated, and the final error estimate *e*(*n*) is the particle scattering pulse signal to be extracted. A block diagram of the principle is shown in Figure 1.

## 3. Variable Forgetting Factor RLS Algorithm

In order to identify the time-varying signal of the small-angle scattering pulse amplitude, the forgetting factor is introduced into the identification algorithm to enhance the tracking ability of time-varying parameters. High-precision parameter estimation for time-varying signals is an important scientific problem for the identification of adaptive systems. To improve the performance of the adaptive identification system, the convergence factor can be introduced into the Hessian matrix of Newton recursive algorithm to estimate the phase, the angular frequency, and the amplitude of multi frequency signals [35]. In order to improve the recognition accuracy of RLS algorithm in static environment and enhance the recognition performance of sparse systems, the *l*_1_ norm constraint can be introduced and combined with the expectation maximization algorithm [36]. In order to simplify the adaptive identification algorithm, Xu et al. divided the original multivariable equation-error autoregressive moving average (EEARMA) model into two subdiscrimination models, and proposed a two-stage generalized extended random gradient algorithm based on the two submodels [37]. EKSIOGLU et al. applied regularization constraints to RLS algorithm to improve the performance of RLS estimating parameters [38]. Ding et al. proposed a high-precision parameter estimation algorithm for linear continuous-time systems based on a parameter decomposition strategy combined with hierarchical theory [39]. With the introduction of the variable forgetting factor RLS algorithm, it can not only ensure the tracking ability of time-varying signals, but also control the steady-state error of the system [33]. By introducing the forgetting factor into the RLS identification algorithm, the fast-tracking ability of the time-varying signal parameters of scattering pulse can be improved and the parameter estimation error can be reduced. However, the difficulty of this identification algorithm lies in the determination of the size of the forgetting factor [29]. The forgetting factor is too small and the parameter estimation fluctuates too much. If the forgetting factor is too large, the ability to track time-varying parameters will be weaker. However, it is not sensitive to noise, and the parameter estimation error is smaller during convergence, so the identification result will be affected [40]. With the increase in data dimensions, both the upper and lower bounds of the parameter estimation error of the least-squares algorithm can tend to the minimum value by choosing the appropriate size of the forgetting factor [29]. In addition, the RLS algorithm that combines decomposition technology with double forgetting factors has a good identification ability for the pseudo-linear system with missing information [41].

In the RLS algorithm, the forgetting factor *λ* is introduced to determine the weight of the data update. The convergence speed, tracking speed, and stability of the algorithm are determined by the size of the forgetting factor [42]. When *λ* is small, the strengthening degree of the new data and weakening degree of the old data are stronger, which can make the previous error work so that the algorithm has a strong tracking ability and convergence speed. When *λ* is larger, the strengthening degree of the new data and weakening degree of the old data are relatively weak. The sensitivity of the algorithm to the error is reduced and the algorithm has high stability. When *λ* = 1, the errors at each time are treated equally. The system has no forgetting ability and degenerates to the ordinary recursive least squares method. When *λ* = 0, the error of the past time is completely forgotten, and only the current error works. In a nonstationary environment, it is desirable that *λ* is small enough. Only a limited number of errors at the nearest moment are required to work, so that the algorithm can quickly track the local trend of the nonstationary signal [43]. On the other hand, it is hoped that in the steady state, *λ* can gradually increase to an appropriate value to reduce the parameter estimation error [44]. The choice of *λ* size should be a compromise between algorithm stability and tracking speed. According to the characteristics of particle pulse signals, a larger value can be used to obtain a smaller system steady-state error in the process of forward scattering pulse signal phase cancellation. This can better eliminate the difference between input signals caused by the direct laser source, the random change of the air molecules, and the stability of the device. When the scattering pulse signal of particles appears, the value of *λ* is small enough, and the sensitivity of the algorithm to the most recent error will increase accordingly. This enables the system to quickly track the local trend of the nonstationary signal to highlight the pulse signal of particles. In the traditional RLS algorithm, the fixed forgetting factor cannot meet these requirements simultaneously. Therefore, a new variable forgetting factor recursive least squares (VFF-RLS) algorithm is designed according to the characteristics of the forward scattering pulse signal of the small-angle OPC.

With the small-angle OPC detection system, by analyzing the signal characteristics we can draw the following conclusions. The forward scattering signals of particles will decay exponentially with the decrease in particle size and the increase in light absorption. When the particle size is large, the scattering pulse signal of particles will be superimposed on the background noise signal peak caused by the light source or air molecules. As the particle size decreases, the pulse amplitude will also decrease until it is completely submerged in the noise. When the OPC detects larger particles, the traditional method of subtracting steady-state noise can obtain better results. When detecting particles smaller than 1 μm, however, the pulse signal becomes extremely weak and difficult to identify. On the other hand, the stray light from the direct laser source and the forward scattering signal of the air molecules often appear in the front region of the time domain. Here, the two signals should ignore the differences to the maximum extent possible for the pair elimination. Even if the scattering pulse signal appears at this point, the algorithm can also realize recognition and extraction. This makes it possible to reduce the steady-state error of the system without losing the ability to quickly track the scattering pulse signal of particles. Based on the characteristics of the abovementioned forward small-angle OPC particle pulse signal, the general idea of the proposed forgetting factor is that the value of the forgetting factor is inversely proportional to the amplitude and time of the error signal. The variation rule is in line with the exponential decay trend. The range and degree of change should be obtained by summarizing the experimental results to obtain the empirical formula and continuously debugging the parameters to obtain the best value. According to multiple sets of experimental data, the following empirical expressions of forgetting factors are given in the designed VFF-RLS algorithm:

(4) $$\left\{\begin{array}{l} \lambda (n)=\lambda_{\min}+{\left(1-\lambda_{\min}\right)}^{\partial (n)}\\ \partial (n)=\exp(\rho e(n)\exp(\alpha n)) \end{array}\right..$$

**Figure 2:**
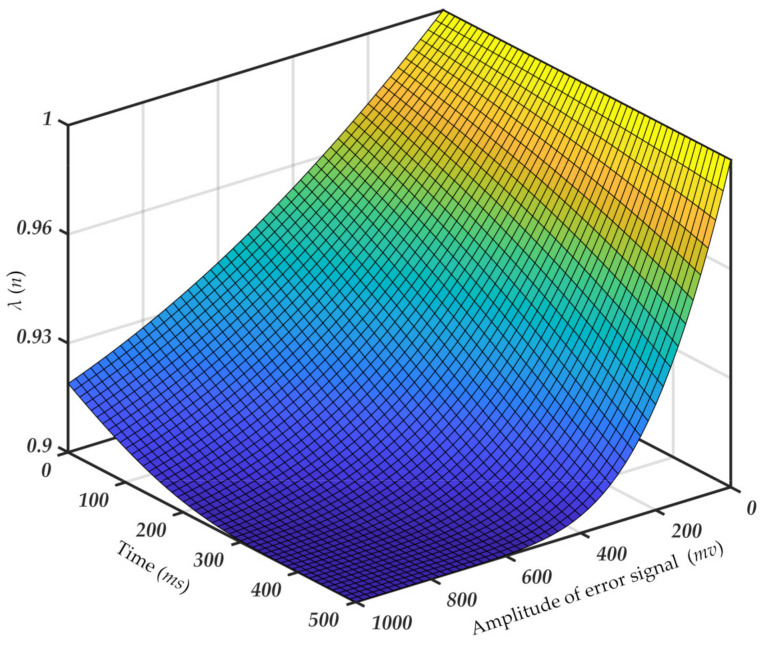
Relation between forgetting, time, and amplitude of the error signal.

The speed and range of the variable forgetting factor *λ*(*n*) can be adjusted by the coefficients *α*, *ρ*, and *λ*_min_. The selection of parameters is determined by the system sampling interval, the input signal amplitude, and the degree of variation. The specific values are obtained by debugging based on experimental data after the system construction is completed. When the difference between the two input signals in the forward scattering part is small, *λ*_min_ and *ρ* can be reduced. When the sampling accuracy of the system increases, α needs to be reduced. According to the summary and debugging of many experiments with this system, the parameters were finally determined as follows: *λ*_min_ = 0.9, *α* = 1.5 × 10^−4^, *ρ* = 0.4. The forgetting factor can vary with the size and time of the error signal, and the change rule is shown in Figure 2.

Figure 2 shows the variation of forgetting factor function with time and error signal amplitude. When the system starts signal acquisition, the direct signal of the light source and the scattering signal of the air molecules appear immediately after the pulse signal, which requires a large forgetting factor to obtain a better cancellation effect. When the amplitude of the error signal is small, the forgetting factor is large, so the algorithm has good stability and can ignore the difference caused by the jitter of the signal waveform. When the error signal is large enough, the forgetting factor decreases, and the system will quickly track the difference between the signals. At the same time, this trend will be more significant as the sampling points move back. As mentioned above, if the particle size is large, the difference between the two input signals around the scattering pulse signal is quite significant. The forgetting factor can achieve a better filtering effect without too much change. If the particle size is small, it is difficult to distinguish the difference between the two input signals at the scattering pulse signal, and even the former will be much smaller than the latter. In this case, the above designed forgetting factor is needed to identify the particle pulse detection signal. Thus, the purpose of adaptively suppressing noise and extracting weak scattering signals from particles is achieved.

## 4. Experimental Results and Analysis

**Figure 3:**
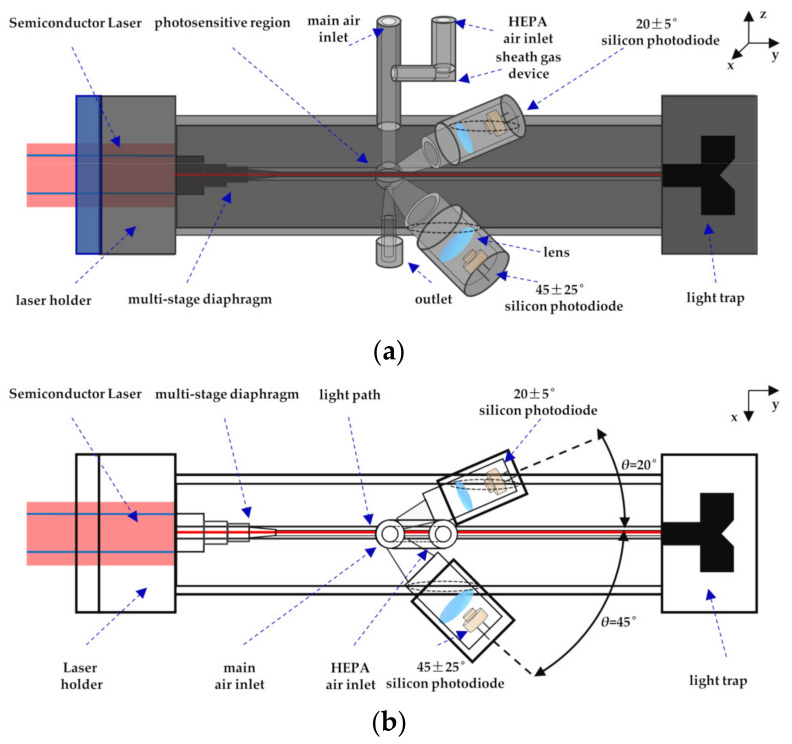
Schematic diagram of the experimental prototype: (**a**) perspective view; (**b**) top view.

**Figure 4:**
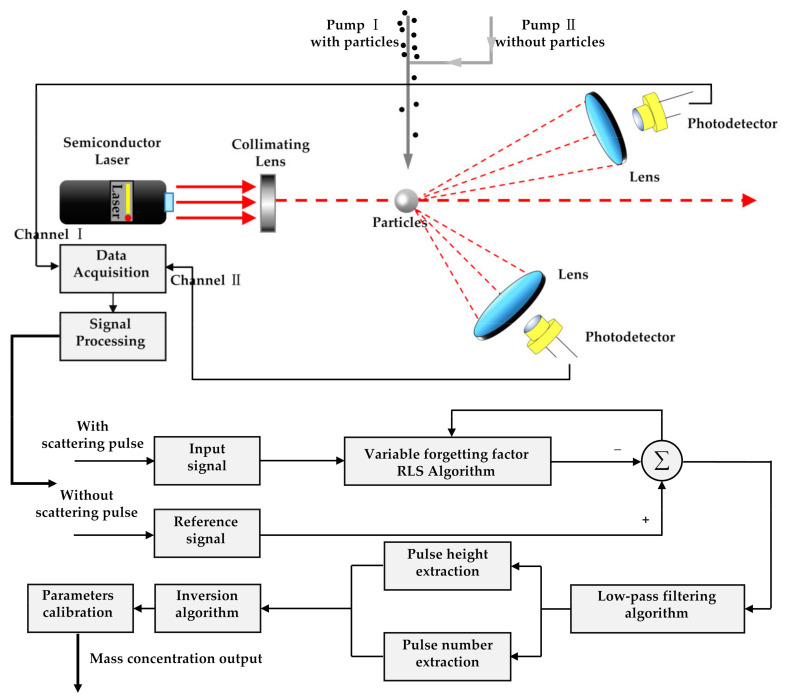
Flow chart of pulse signal processing.

To evaluate the feasibility of the proposed method and the effect of adaptive filtering, an experimental prototype of the small-angle OPC detection of particles was designed, as shown in Figure 3. The light source is a 100 mw semiconductor laser with a wavelength of 650 nm, a beam diameter of 5 mm, and a divergence angle of 8 mrad. The light beam passes through the optical collimation system and multi-stage diaphragm to reduce the halo, and then enters the photosensitive region. The scattering light generated by the interaction of the laser beam and the particles is received by the forward 20° and side 45° silicon S2386-44K photodiodes (Hamamatsu Inc., Shizuoka, Japan). An angle of 20° was used to detect the scattering pulse signal of particles, and an angle of 45° was used to detect the photometric signal of particles. A Submicrometer Monodisperse Aerosol Generator (TSI Inc., Shoreview, MN, USA) was used to evenly suspend the standard size particles in the smoke box, and the atomization volume was 3 L/min. The monodisperse standard particles reagent selected for the aerosol generator was polystyrene microsphere suspension liquid (0.2 μm: 3K-200, 0.5 μm: 3K-500, 1 μm: 3K-1000, Thermo Fisher Scientific Inc., Waltham, MA, USA). The standard particle density was 1.05 g/cm^3^ and the number concentration was 1 × 10^9^/mL. The standard particles were diluted to 1 × 10^3^/mL with deionized water and then atomized with an aerosol generator. A dryer was equipped at the outlet to remove the water in the particles. The particles in the smoke box were sent to the photosensitive region of the prototype by air pump. The inlet air path of the designed small-angle OPC prototype is divided into two parts. A portion of the particles from the external environment are directly extracted by air pump 1 and sent into the photosensitive area. The other part is connected to the sheath gas system by air pump 2, and the particle-free air filtered by the high-efficiency particulate air (HEPA) filter is passed into the photosensitive area to dilute the particle concentration. During the measurement period, air pump 2 is working. When air pump 1 stops air intake, the signal measured by the light detector is the reference signal without particles. When air pump 1 is working, the signal measured by the light detector is the detection signal with particles. The reference signal without particles and the detection signal with particles received by the photodetector are, respectively, sent to the data acquisition card (ART Inc., China). The data are transferred to the virtual host computer LabVIEW in real time for storage and online processing. The signal processing process is shown in Figure 4. The two signals are used as the input parameters of the designed VFF-RLS, and they cancel each other through adaptive adjustment, making the scattering pulse signal of particles irrelevant.

**Figure 5:**
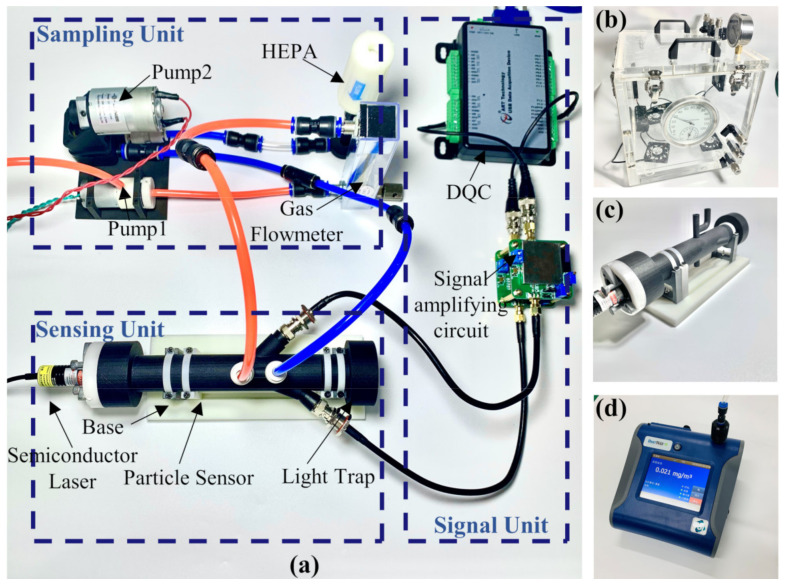
Small-angle OPC measurement system: (**a**) overall composition of the system, (**b**) smoke box, (**c**) detection prototype, and (**d**) DustTrack 8530.

The experimental setup is shown in Figure 5. When detecting large-sized particles, the pulse amplitude of scattering signal is relatively large. The traditional method of directly subtracting the zero-drift value can achieve better results. However, as the particle size gradually decreases, the scattering pulse signals are gradually submerged in the noise, and it is almost impossible to distinguish the scattering pulse height. In practice, the ability of the traditional small-angle OPC to extract smaller particle pulses is greatly reduced due to the sampling accuracy limitation and device noise, and large ripple signals often appear. The adaptive filtering method proposed in this paper can solve these problems.

**Figure 6:**
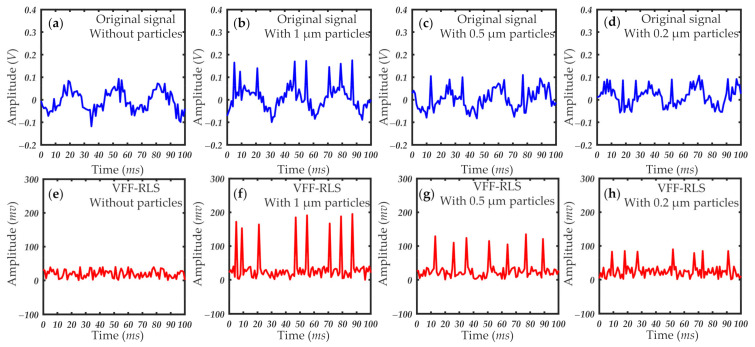
Original detection signal and VFF-RLS filtered signal with and without particles.

According to the analysis in Section 2, the signal sources received by the photodetector mainly include the following five types: the direct light from the laser source or the reflected light from the inner wall, the scattering light from the air molecules, the natural light from outside, the system background electrical noise, and the scattering light from the particles. Due to the high-performance light absorbing material used in the shell, the external transmitted light can be ignored. This is in order to prove that the extracted pulse signal is indeed caused by the scattering light of the particles, and not caused by other stray light or system noise. It is necessary to conduct a set of control experiments on the signals received by the photodetector with and without particles. After the optical mechanical system is built, the first four kinds of signal sources have been relatively stable without changing the laser, optical detector, amplification circuit and shell. The analysis shows that the first four kinds of signals will not change with the time and location. In order to obtain the control group without the effect of particle scattering, we used the signal without particle scattering filtered by HEPA as the input signal and reference signal of the proposed VFF-RLS algorithm. The original detection signal and filtering signal without particles are shown in Figure 6a,e. In order to obtain the control group with particle scattering under different particle sizes, the signal with particle scattering was used as the input signal, while the signal without particle scattering was used as the reference signal of VFF-RLS algorithm. The original detection signal and filtering signal with particles are shown in Figure 6b–d,f–h. As can be seen from Figure 6, under the condition that other factors such as the first four signal sources remain unchanged, the signal of the photodetector has changed significantly in the presence of particles. According to the filtering results with or without particles in Figure 6, the VFF-RLS algorithm we proposed can effectively suppress noise, and the extracted pulse signal is indeed caused by particles scattering.

**Figure 7:**
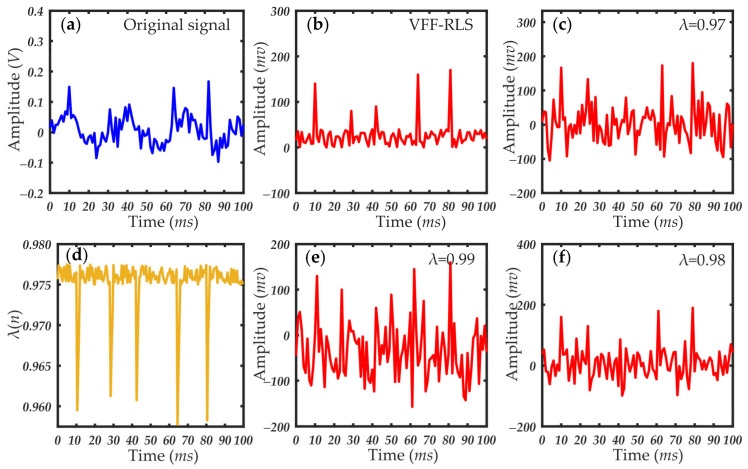
Particle scattering signal and filtering results (particle size: 1 μm): (**a**) original scattering signal; (**b**) particle scattering signal processed by the VFF-RLS algorithm; (**d**) change curve of forgetting factor; (**c**,**e**,**f**) results of RLS algorithm with fixed forgetting factors, which are 0.97, 0.99, and 0.98, respectively.

**Figure 8:**
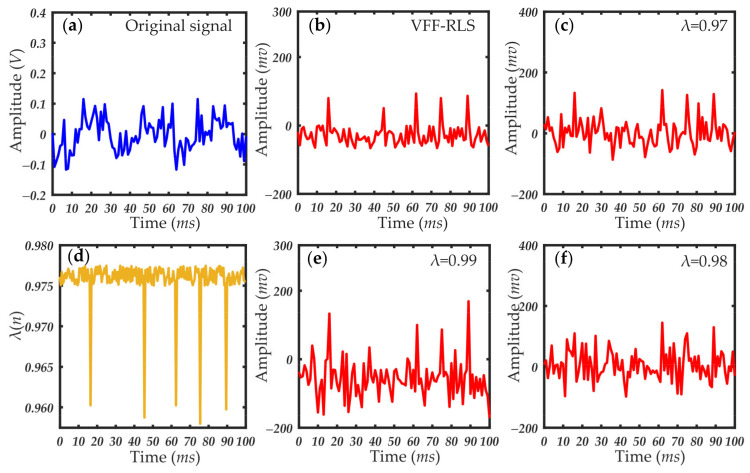
Particle scattering signal and filtering results (particle size: 0.5 μm): (**a**) original scattering signal; (**b**) particle scattering signal processed by the VFF-RLS algorithm; (**d**) change curve of forgetting factor; (**c**,**e**,**f**) results of RLS algorithm with fixed forgetting factors, which are 0.97, 0.99, and 0.98, respectively.

**Figure 9:**
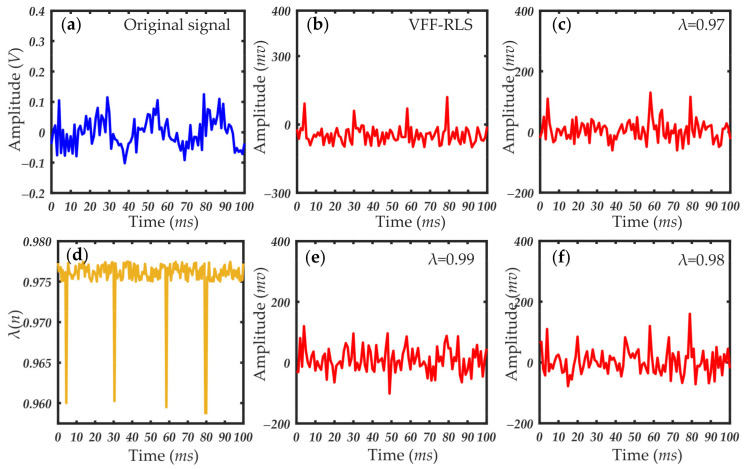
Particle scattering signal and filtering results (particle size: 0.2 μm): (**a**) original scattering signal; (**b**) particle scattering signal processed by the VFF-RLS algorithm; (**d**) change curve of forgetting factor; (**c**,**e**,**f**) results of RLS algorithm with fixed forgetting factors, which are 0.97, 0.99, and 0.98, respectively.

Figure 7, Figure 8 and Figure 9 show the results of the small-angle OPC detection signals and extracted forward scattering pulse signal of particles of different sizes. As seen in Figure 7, Figure 8 and Figure 9, the experimental data processed with the variable forgetting factor RLS algorithm accurately extracted the scattering pulse signal of the particles regardless of their size. The pulse signal resolution was greatly improved, compared with that before processing. During the processing, the change curve of the forgetting factor was consistent with the previous algorithm design ideas. When the scattering pulse signal of the particles appeared, the forgetting factor had a large mutation. The smaller value of the forgetting factor facilitated faster tracking of the signal. At other times, the value of the forgetting factor was larger and remained stable. This was conducive to signal cancellation, which effectively suppressed the forward scattering noise, thus highlighting the scattering pulse signal. When compared to the processing results of the RLS algorithm, using the same value of the forgetting factor for different particle size pulse signals returned very different results. Whether the forgetting factor took any fixed value in the variation range, the ideal effect of pulse signal extraction could not be achieved. This further verified the necessity of improving the fixed forgetting factor of the common RLS algorithm. The applicability of the variable forgetting factor proposed in this paper in extracting forward scattering pulse signals from small-angle OPC was proved.

## 5. Discussions

When using the variable forgetting factor RLS algorithm to extract the forward scattering pulse signal of particles, in addition to the forgetting factor, the size of filter order *L* also affected the processing result. The size of the forgetting factor determines the degree of cancellation between the input signal and the reference signal. The size of the filter order determines the accuracy of the filter operation. The filter order *L* can be understood as the degree of subdivision of the detection signal, that is, the length of useful information input to the system during each iteration of the calculation. Generally, the larger *L*, the greater the amount of information used in the iterative computation of the system, and the calculation amount and calculation time of the proposed algorithm will increase correspondingly, and the signal processing will be more accurate, and the stronger the fitting ability of the filter. Conversely, if a smaller filter order is set, the calculation amount and calculation time will decrease significantly, but the measurement accuracy will also decrease accordingly. Therefore, the filter order *L* will affect the filtering accuracy and the calculation time of the measurement results. For this system, the higher the filter order, the better the filtering effect, and the higher the resolution of the small-angle scattering pulse of particles, but the system operation time will increase accordingly. The lower the filter order, the lower the system operation time will be, but the filtering effect and accuracy will also become worse. Too small filter order will even make the signal distortion. If we blindly pursue the filtering effect and the operation accuracy, and thus simply increase the filtering order, it will slow down the system response speed and increase the system running time. In addition, to extract the scattering pulse of a fixed particle size, the filter order will not improve the resolution of the small-angle scattering pulse of particles when it is high to a certain numerical range. The long system running time cannot meet the requirements of the proposed detection system for automatic real-time operation and online processing. In the actual extraction of small-angle scattering pulse of particles, the choice of the VFF-RLS filter order needs to balance filtering effect and calculation time. An appropriate filter order range can make the algorithm not only complete the calculation quickly, but also reduce the system background noise and improve the resolution of the scattering pulse of particles. That is, the algorithm can process the scattering pulse signals of larger particle size more quickly while also being able to process the scattering pulse signals of smaller particle size without distortion.

**Figure 10:**
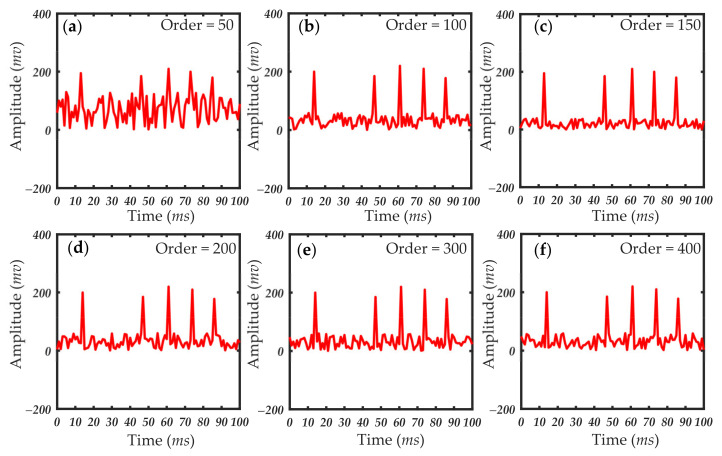
Results of 1 μm particles under different filter order settings: (**a**) filter order = 50; (**b**) filter order = 100; (**c**) filter order = 150; (**d**) filter order = 200; (**e**) filter order = 300; (**f**) filter order = 400.

**Figure 11:**
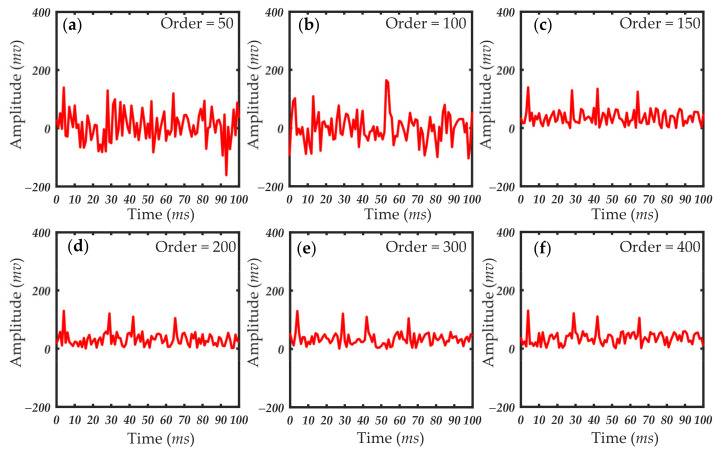
Results of 0.5 μm particles under different filter order settings: (**a**) filter order = 50; (**b**) filter order = 100; (**c**) filter order = 150; (**d**) filter order = 200; (**e**) filter order = 300; (**f**) filter order = 400.

**Figure 12:**
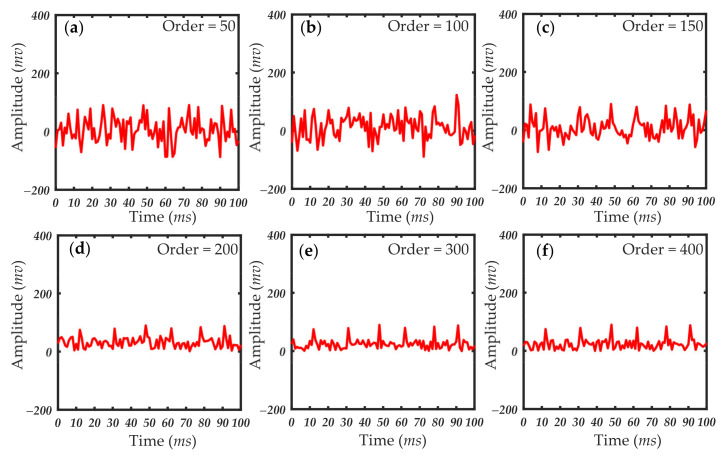
Results of 0.2 μm particles under different filter order settings: (**a**) filter order = 50; (**b**) filter order = 100; (**c**) filter order = 150; (**d**) filter order = 200; (**e**) filter order = 300; (**f**) filter order = 400.

In practical applications, the approximate value of the filter order is estimated first. Then, through experiments, the length of the filter order is gradually reduced without any significant reduction in filter quality. Finally, the filter order range of the selected filter is determined. Figure 10, Figure 11 and Figure 12 show the processing results of 1, 0.5 and 1 μm particles under different filter order settings. The size of the filter order of the RLS algorithm had little effect on the processing results when detecting particles of a larger size. The smaller filter order cannot achieve the ideal filtering effect when the particle size is small. When the scattering pulse signal is relatively weak, the order of the filter should be increased. When the scattering pulse signal is relatively strong, the order can be reduced to shorten the processing time.

It can be seen in Figure 10, Figure 11 and Figure 12 that the filtering effect of different particle sizes with the filter order around 300 has tended to be ideal. Therefore, the filter order range of this system is between 200 and 300. In this filter order range, the scattering pulse signal of 0.2 μm particles can be extracted without distortion, so that it has better resolution in extracting scattering pulse signal with small particle size of 0.2 μm. At the same time, it can meet the rapid extraction of scattering pulse signal with larger particle size of 1 μm. The accuracy of the data measured by different detection environments and sampling devices was different, and the optimal range of the filter order was then adjusted to the specific conditions of the different systems. Thus, the order of the filter should be determined according to the performance and environment of the actual system. As the detection particle size signal becomes weaker, the value needs to be traded off between filtering effect and calculation time.

## 6. Conclusions

The proposed variable forgetting factor RLS algorithm can self-adapt to the random changes in atmospheric environment, particle scattering characteristics, and system noise. It can deal with the interference caused by the forward scattering of the air molecules and direct light source. The change rule of the improved forgetting factor is related to the scattering characteristics of the atmospheric particles. Thus, the method has a good tracking ability for the scattering pulse signals of different particle sizes and can converge quickly when the scattering pulse appears. The actual particle detection experiment proved that the system can detect particles smaller than 1 μm in real time in the atmospheric environment. Obtaining the appropriate reference information for the atmospheric environment, increasing the speed of weak pulse signal extraction, and reducing the interference from prior information differences in the algorithm processing results is the focus of future work.

## Data Availability

We did not report any data.
